# Eliciting health state utilities for Aromatic L-amino Acid Decarboxylase (AADC) deficiency: a UK vignette study

**DOI:** 10.1186/s41687-021-00403-0

**Published:** 2021-12-11

**Authors:** Adam B. Smith, Andria Hanbury, Katharina Buesch

**Affiliations:** 1 York Health Economics Consortium Ltd., University of York, Enterprise House, Innovation Way, York, YO10 5NQ UK; 2 PTC Therapeutics, Inc., 100 Corporate Court, South Plainfield, NJ 07080 USA

**Keywords:** AADC deficiency, Vignettes, Time trade-off, Standard gamble

## Abstract

**Purpose:**

The aim of this study was to generate health state utilities for aromatic L-amino acid decarboxylase (AADC) deficiency, a rare genetic, lifelong neurogenerative condition predominantly manifesting in young infants.

**Methods:**

Participants were presented with health state vignettes. These had been previously developed based on published literature, clinician input, interviews with parents of AADC deficiency patients and expert opinion. A total of 5 health state vignettes were presented: bedridden, head control, sitting unsupported, standing with assistance and walking with assistance. Health state utilities (HSU) were elicited using time-trade off (TTO; 10-year time horizon) and the standard gamble (SG). The vignettes were completed online by panel participants drawn from a representative sample of the United Kingdom residential population.

**Results:**

A total of 1598 participants completed the vignettes. Around 21% had incongruent responses (higher utilities for the bedridden compared to walking health states). Incongruent responses were associated with shorter task completion times, gender and parental status. These responses were removed from the analysis. Health state utilities (HSU) increased correspondingly as health states improved for both the TTO and SG. The mean HSU (standard deviation) for the TTO task were: bedridden state 0.49 (0.34); head control 0.54 (0.33), sitting unsupported 0.63 (0.31); standing with assistance 0.68 (0.31); and walking with assistance 0.73 (0.31). For the SG, mean health state utilities were: 0.56 (0.28), 0.57 (0.27), 0.67 (0.24), 0.70 (0.24), and 0.75 (0.25), respectively.

**Conclusion:**

Health state utilities were derived for AADC deficiency through a vignette study. These will be used for a cost-effectiveness model of an AADC deficiency treatment.

**Supplementary Information:**

The online version contains supplementary material available at 10.1186/s41687-021-00403-0.

## Introduction

Aromatic L-amino decarboxylase (AADC) deficiency is a genetic condition, which typically presents in early infancy. It is a rare condition with only around 150 reported cases worldwide [1, 2]. Common symptoms are hypotonia, developmental delay, and movement disorders, including oculogyric crises [1, 2]. Patients with the severe phenotype will be bedridden for life [2], will not achieve developmental milestones and will be fully dependent for care. Severe phenotypes may also be associated with a significant mortality risk [3]. Even cases with the mild phenotype will display a degree of developmental delay, cognitive disability and require assistance with walking. Although AADC deficiency clearly has the potential to significantly impact on patients’ health-related quality of life (HRQoL), the nature and rarity of AADC deficiency means it is difficult to derive robust health state utilities from either the child or their parent/caregiver [4, 5]. Not only are HRQoL assessments necessary to evaluate the impact of AADC deficiency on the child, these evaluations in the form of health utilities are often required in economic evaluations of therapeutic interventions. Health utilities are societal-based preferences for a given health state and are rated on a scale from 0 to 1, where 0 represents “death” and 1 “perfect health”. States worse than death are also possible. In many cases health utilities may be obtained through patient-reported outcome measures (PROs), such as the EuroQol 5-dimension (EQ-5D) [6–8] and the Health Utilities Index (HUI3) [9]. However, in rare diseases and in particular paediatric populations, this process becomes more problematic, as for obvious reasons the child is unable to complete the instruments.

Other approaches for generating health state utilities need therefore to be considered. For instance, one approach may be to derive proxy-ratings from clinicians using preference-based measures such as the EQ-5D or HUI3 [10]. In these studies clinicians may be asked to rate either hypothetical cases (using descriptions or “vignettes”) or actual case studies. Typically, the number of clinicians involved in these studies is small. Furthermore, there are inherent issues in proxy-ratings (for both clinicians and parents and caregivers) in the form of (unwitting biases) [11]. Other methods for deriving health state utilities include time trade-off (TTO) and standard gamble (SG) tasks [12]. In both methods, similar to the hypothetical clinician case studies, participants (drawn usually from representative general population samples) are presented with health state descriptions in the form of vignettes. In the TTO, participants are told they have a few years of life to be spent in the health state described; these life-years may be traded off in return for spending the remaining years in full health. For the SG, participants are informed there is a cure for the condition described, however there is a risk that this will fail leading to immediate death. Participants are asked to select the level of risk for a specific treatment failure they are willing to accept in return for perfect health. Responses on both tasks can be converted into health state utilities. These approaches, including those where preference-based measures included in the TTO and SG tasks have been used to derive health state utilities for rare conditions [13]. To the best of the authors’ knowledge there have been no published studies describing health (state) utilities for patients with AADC deficiency. The aim of this study, therefore, was to derive health state utilities for AADC deficiency using TTO and SG vignettes. The results of the study will be used as inputs to an economic model evaluating the cost-effectiveness of gene therapy for AADC deficiency.

## Methods

### Sample

Respondents were recruited from a panel maintained by a third party (Qualtrics, Provo, USA). The sample was selected to be broadly representative of the residential UK adult population. The sample was stratified on the basis of data published by the UK Office for National Statistics (www.ons.gov.uk). For country, England represented 85% of the sample, Scotland 10%, Wales and Northern Ireland 2.5% each; gender was stratified to ensure a roughly 50:50 representation of males and females; age was stratified into approximately three equal categories (18–34, 35–54 and 55 + years of age); and finally, education was stratified to ensure around 75% of the sample had completed lower and higher secondary education and the remainder graduate education. Basic socio-demographic details (age, biological sex (male/female/prefer not to say), parental status, and country of residence), were collected from respondents and used to screen for eligibility. Respondents were eligible to participate provided they were a UK resident and aged ≥ 18 years. Parents and caregivers of children with life-threatening or life-limiting conditions were not eligible to participate in order to reduce any potential bias. The study was conducted on an online platform and participants completing the study received a nominal incentive (redeemable points) reflective of the time required to complete the study. The study was submitted for review to the University of York’s Health Sciences Research Governance Committee and received ethics approval on 20 March 2020, and was conducted in accordance with the Declaration of Helsinki. An initial sample size of N = 1000 was estimated to be sufficient to allow robust parameter estimation and allow subgroup analyses.

### Vignettes

The development of the vignettes has been described in detail elsewhere [14]. In brief, the vignettes were initially developed from a pragmatic literature review; a review of case stories provided online from AADC deficiency support groups; an advisory board with parents and caregivers of children with AADC deficiency; and an advisory with physicians of patients with AADC deficiency. In the final stage of the process the vignettes were reviewed by parents and caregivers of children with AADC deficiency and physicians. A total of five health state vignettes were developed reflecting motor and developmental milestones. These were based on an ongoing clinical trial investigating gene therapy for AADC deficiency (NCT02926066) and correspond to the economic model being developed to evaluate this therapy. The five health states were: 1. the base health state (“bedridden” or untreated AADC).; 2. “head control”; 3. “ability to sit unaided”; 4. “standing with support”; and 5. “walking with assistance”. An additional vignette was developed in order to capture parental/caregiver quality of life. The vignettes also included descriptions of the main symptoms of AADC deficiency: oculogyric crises, feeding ability, cognitive impairment and screaming. The vignettes were designed to reflect a gradual global improvement in milestones and symptoms moving from the “bedridden” through to the “walking with assistance” health state. An example of one of the vignettes is shown in Box 1.

**Box 1:** **Example health state**

Health State Description	
Bedridden (Worst Health State)	
Imagine that you are a parent or caregiver of a child with a severe medical condition. This medical condition means that:	
Your child is bedridden and unable to move by themselves. This means that your child is unable to lift and control their head, crawl, sit, or stand	
They are unable to feed themselves and may need to be fed through a tube	
They have very poorly developed muscle tone meaning their body, arms and legs are very floppy. This means your child is unable to grasps or hold onto things	
Your child will also frequently experience painful muscle spasms, and their arms and legs may move involuntarily with sudden jerking or twisting	
Your child screams constantly throughout the day and night	
Your child experiences something called an “oculogyric crisis”. This is where their eyes rotate or roll in unusual ways, similar to an epileptic seizure. These may last for up to several hours, several times a day	
Your child will have problems sleeping	
Your child will have severe abdominal problems, such as constipation or diarrhoea	
They will be extremely irritable and agitated	
Your child is unable to follow objects with their eyes and unable to recognise and interact with people. They can understand simple words but are not able to speak	
Other symptoms include severely blocked nose (“nasal congestion”), which may lead to serious chest infections, as well as excessive drooling, excessive sweating and extreme tiredness	

### Procedure

The online survey was tested in a number of rounds of piloting (approximately 50 participants per round). This revealed that a proportion of (36.8%) of participants in the pilot study were providing incongruent responses. Incongruent is defined here as rating the “worst” health state (“bedridden”) higher than the “best” health state (“able to walk”) on the TTO task.^1^ An initial analysis demonstrated that participants with incongruent responses were completing the study within a shorter time period (approximately 5.5 min) compared to the other participants (approximately 7 min). As a result, a time delay was imposed ensuring that the study could not be completed in less than 6 minutes. Furthermore, the sample size requirement was increased to 1500 to ensure as much as possible that a minimum of 1000 congruent responses would be collected.

Participants were shown an informed consent form in the initial part of the study, describing the nature of the study, as well as the study sponsor. This form also contained a warning regarding the descriptions of AADC deficiency in the vignettes as this is by nature a severe medical condition. Participants were also informed that they were free to withdraw from the study at any stage. Once the participant had provided their consent a screening question was presented to ensure that any parent or caregiver of a child with a potentially life-threatening or life-limiting condition was screened out of the study. The participants were then asked to provide their home nation in the United Kingdom (England, Scotland, Wales or Northern Ireland); their age; biological gender (male, female, prefer not to say), highest level of education (lower secondary up to age 16, higher secondary up to age 18, undergraduate, postgraduate).

Participants were subsequently provided with an explanation of the response format for the TTO and SG and were taken through the process in order to familiarise themselves with the rating system. The participants were informed that they would be asked to imagine themselves as the parent or caregiver of the child described in each of the five vignettes. Each vignette provided a description of a hypothetical child with AADC deficiency (Box 1). The degree of severity of the symptoms described in the vignettes (e.g. oculogyric crises, sleeping problems, motor skills, etc.) was varied across the five vignettes, so that the severity decreased as the health state improved. The TTO time period for each vignette was 10 years, i.e. participants were told the child had 10 years of life to live in the health state described. In the TTO, participants were asked to indicate how much of the child’s life they were willing to trade in order for the child to live the remaining years in full health. The 10-year time period is the most commonly applied time horizon for TTO tasks, however, its use has been criticised in paediatric samples [15] where the length of time may be considered to be too great. This may be germane where developmental milestones, for instance, are being met over shorter periods of time, and where 10 years would therefore not be a realistic time period over which to assume an unchanging health state. However, the AADC deficiency case studies described in the literature report patients in bedridden states (from birth) who have remained in those states for lengthy periods of time extending well beyond 10 years. For instance, 5 patients described in the literature had been bedridden for an average 12 years. The 10-year time horizon for the TTO task (describing unchanged health states) is therefore supported by case studies from the literature.

For the SG, participants were told there was a cure available to treat the child resulting in perfect health, however, that there was also a risk the treatment could fail leading to the immediate death of the child. Participants were asked to indicate the level of risk of immediate death they were willing to accept. Risk was rated on a scale of 0 to 100 (the higher value reflecting the greater degree of risk participants were willing to accept).

A slider bar was used for both tasks: for the TTO tasks moving the slider in either direction presented the participants with the number of years traded (x) on one side of the bar against the remaining years in full health (10-x) on the other side. A similar design was used for the SG task, where the level of risk participants were willing to accept was shown against the chance of the treatment succeeding. The point of indifference for the TTO and maximum risk of death were captured (visually) in this way.

The bedridden health state was presented first; the other health states were subsequently presented in a random order. Once participants had completed these health states, they were presented with a further vignette describing a child with AADC deficiency and asked to provide a rating for parental or caregiver quality of life on a visual analogue scale ranging from 0 (“worst possible quality of life”) to 100 (“best possible quality of life”).

### Statistical analysis

The data were collected on an MS Excel spreadsheet and transferred into SPSS (IBM SPSS Statistics, version 25). Further analysis was undertaken using Stata/SE (StataCorp, 2021). The health state utilities were derived by subtracting the participants’ response from 10 (TTO) and 100 (SG), and dividing the result by 10 and 100, respectively. This meant that the highest possible health state utility value was 1 (perfect health) and the lowest 0; in other words, none of the health states could be rated as worse than dead.

Descriptive statistics (mean, standard deviation (SD) and 95% confidence intervals (95%CI)) were generated for the utilities (TTO, SG) for each health state and across the sociodemographic categories. Means were also produced for parental / caregiver quality of life. Independent samples t-tests were used to evaluate differences between participants with congruent with incongruent responses in terms of study completion time and age. Chi-squared tests were used to determine any differences between these two groups in terms of gender, parental status and education level. A one-way within-subjects analysis of variance (ANOVA) was used to evaluate differences in overall health state utilities between the vignettes (both TTO and SG) and to explore differences in health state utilities and parental/caregiver quality-of-life across the vignettes by gender, parental status and education level.

## Results

### Participants

A total of 1598 participants completed the study of which 330 (20.7%) were incongruent responses (mean utility for the “walking” health state < “bedridden” health state utility (HSU). As a further quality check, the number of participants rating the “walking” health state less than the non-walking health states (where the mean utility for bedridden < head control < sitting < standing) was also assessed. This represented 6.2% of the overall sample (N = 99); however, all of these participants also fell into the “main” incongruent category (bedridden HSU > walking HSU). Participants with incongruent responses completed the tasks in a shorter period of time (631 s, standard deviation (SD) 678 s; compared to 810 s, (SD 2156) for those with congruent responses). However, this difference was not statistically significant (t(1596) = 1.49, *p* = 0.14; nor were there any significant differences in terms of age (42.7 years, SD 17.3 years and 44.2 years, SD 16.5, respectively) (t(1596) = 1.43, *p* = 0.15). Furthermore, there were no statistically significant differences compared to those with congruent responses in terms of education (X^2^_(3)_ = 1.50, *p* = 0.47. There were, however, differences in terms of gender (of the incongruent responses 52.9% were female, 47.1% male, X2(1) = 5.34, *p* = 0.021) and parental status (X^2^_(1)_ = 8.83, *p* = 0.003) (65.2% not a parent versus 34.8%). These incongruent responses were subsequently removed from the analysis. Table 1 provides a breakdown by sociodemographic details. There were proportionally more females (59.8%) in the sample. Average age was 44.2 years (SD 16.47 years). A minority of participants were parents (26.6%), however none were parents of children younger than 16 years of age.

**Table 1:** Sociodemographic details

	Gender, N (%)	Age (standard deviation)
Female	758 (59.8)	41.3 (16.17)
Male	506 (39.9)	48.7 (15.89)
Prefer not to say	4 (0.3)	24.3 (6.40)
Overall	1268	44.20 (16.47)
Education	Frequency	Percent
Lower secondary education	408	32.3
Higher secondary education	647	51.0
Undergraduate degree	162	12.8
Postgraduate degree	51	4.0
*Parent*		
No	931	73.4
Yes	337	26.6
*Location*		
England	1075	84.8
Scotland	132	10.4
Wales	32	2.5
Northern Ireland	29	2.3

### Overall health state utilities

The overall health state utilities are shown in Table 2. For the TTO utilities there is a corresponding increase across the health states as these improve (i.e. as symptom severity decreases and motor skills improve). This is particularly marked moving from “head control” to “sitting unaided” with a utility difference of just under 0.10; the remaining difference between adjacent states was aro und 0.05. These differences were statistically significant (F(4,5068) = 448.38, *p* < 0.0001). The 95% confidence intervals (CIs) suggest statistically significant differences between both adjacent health states, as well as the remaining contrasts (*p* = 0.05). There was an overall effect for the SG utilities as well (F(4,5068) = 357.53, *p* < 0.0001). There was effectively no difference in utility values for the “bedridden” and “head control” states, although there was a noticeable corresponding increase from the latter health state onwards. The difference between utility values for “head control” and “sitting unaided” as well as the other contrasts were statistically significant (*p* = 0.05). The utilities for SG were greater than the TTO utilities for each corresponding health state, however these differences were relatively small with the largest difference 0.07 for the bedridden state and the smallest difference for the walking with assistance health state, 0.02. The SG utilities ranged from 0.5626 to 0.7489, and the TTO utilities ranged from 0.494 to 0.7279. The overall mean utilities by task are shown in Table 2.

**Table 2:** Overall health state utilities

Mean (SD) utilities	Bedridden	95%CI	Head control	95%CI	Sitting unaided	95%CI	Standing with assistance	95%CI	Walking with assistance	95%CI
TTO	0.494 (0.3429)	0.4751 to 0.5129	0.5369 (0.3255)	0.519 to 0.5548	0.6312 (0.3099)	0.6141 to 0.6482	0.6755 (0.3073)	0.6755 to 0.6925	0.7279 (0.3052)	0.7111 to 0.7447
SG	0.5626 (0.2843)	0.5469 to 0.5783	0.5729 (0.2732)	0.5578 to 0.5879	0.6713 (0.2477)	0.6577 to 0.685	0.7099 (0.2397)	0.6967 to 0.7231	0.7489 (0.2464)	0.7353 to 0.7625

*Key: SD, standard deviation; TTO, time trade-off; SG, standard gamble; 95%CI, 95% confidence interval

### Health states by sociodemographic categories

#### Gender

Due to the low numbers of participants who preferred not to record their gender (N = 4) these respondents were removed from the analysis. Although there was a corresponding increase across the vignettes for both groups, female participants rated all of the vignettes slightly higher than males with a difference between the two groups ranging from 0.0195 (“walking with assistance”) to 0.0398 (“head control”). The range for females was 0.5072 (SD 0.3418) to 0.7358 (SD 0.3073) for the TTO task; for males this was 0.4747 (SD 0.3432) to 0.7163 (SD 0.3018). Only the differences between groups for “head control” and “sitting” were statistically significant (t(1262) = 2.14, *p* = 0.033, and t(1262) = 2.23, *p* = 0.026, respectively).

A similar pattern was observed for the SG task. The range was 0.5743 (SD 0.2803) to 0.7603 (SD 0.2489) for females and 0.5432 (SD 0.2895) to 0.0.7316 (SD 0.2424) for males. The largest difference here was for “head control” 0.4472 and the smallest for “walking with assistance”, 0.02872. These differences were all statistically significant with the exception of the “bedridden” health state: “head control” (t(1262) = 2.86, *p* = 0.004); “sitting”, (t(1262) = 2.13, *p* = 0.033); “standing”, (t(1262) = 2.87, *p* = 0.004); and, “walking with assistance”, (t(1262) = 2.03, *p* = 0.042).

### Parental status

The health state utilities by parental status indicated that with the exception of the “sitting” health state parents rated the health states slightly higher than respondents who were not parents. The range for parents was 0.5025 (SD 0.3445) to 0.7305 (SD 0.3153) for the TTO task; for participants who were not parents this was 0.4909 (SD 0.3425) to 0.7270 (SD 0.3153). The difference ranged from 0.004 to 0.0307. None of these differences were statistically significant.

For the SG task, again parents rated the health states slightly better with the exception of “standing”. The range was 0.5965 (SD) to 0.7570 (SD 0.2381) for parents and 0.5503 (SD 0.2884) to 0.7460 (SD 0.2494) for those who were not parents. Statistically significant differences were only found for the SG for the “bedridden” health state (t(1266) = 2.57, *p* = 0.01).

### Education

For the health state utilities by education level the undergraduate and postgraduate responses were combined (due to the number of these participants relative to the other two groups). Although there was an increasing trend evident across all 3 groups as health states improved, those respondents with lower and upper secondary level education tended to have similar utility values, whereas university educated respondents tended to have higher utility values compared to the other two groups. These differences were statistically significant for the “head control” (F(2, 1265) = 3.60, *p* = 0.028; “sitting” (F(2,1265) = 3.48, *p* = 0.031; “standing with assistance” (F(2, 11,265) = 4.36, *p* = 0.013) and “walking with assistance” (F(2,1265) = 4.20, *p* = 0.015) health states for the TTO only.

### Parental/caregiver quality-of-life

Participants who preferred not to record their gender rated parental / caregiver HRQoL much higher than the other 2 groups (Table 3). The difference in HRQoL between males and females was not statistically significant. Participants who were parents rated HRQoL higher than those who were not parents, although this was not statistically significant. Finally, there was a negative gradient in terms of HRQoL and education, with university educated participants rating parental/caregiver HRQoL lower than the other two groups. This difference was, however, not statistically significant (*p* = 0.074).

**Table 3:** Health-related quality-of-life by gender, parental status and education

Gender	Mean	Std. Deviation	F (df)	*p*
Female	34.77	26.40	3.20 (1,1262)	0.074
Male	37.54	27.85		
Prefer not to say	62.75	28.93		
*Parent*				
No	35.12	26.80	3.38 (1,1266)	0.066
Yes	38.28	27.64		
*Education*				
Lower secondary	37.99	28.53	2.61 (2,1265)	0.074
Higher secondary	35.72	26.74		
University	32.83	24.78		
Total	35.96	27.05		

## Discussion

The aim of this study was to derive health state utilities for patients with AADC deficiency. The results demonstrated a corresponding increase moving from the most to the least severe health states for both the TTO and SG tasks. For the TTO there was a mean difference of around 0.05 utilities between health states with the exception of the “sitting unaided” health where the difference was greater (0.10 utilities difference from “head control”). This was also reflected in the SG tasks suggesting that this might be a key milestone for participants. The SG utilities were marginally higher than the corresponding TTO health state utilities with little separation between the two worst health states. This commonly occurring phenomenon has been reported since the early literature [16, 17] and may be attributable to participants’ difficulty appraising and processing probabilities in the SG [18]. In addition to the overall health state utilities, it was also observed in the analysis by sociodemographic groups. Differences were also observed for the latter, although not all were statistically significant. For instance, higher health state utilities tended to be provided by female participants. Parents were more likely to rate health states slightly better than non-parents. Nevertheless, taken together the results from this large, broadly UK representative sample showed an increase in the utility values as the health states improved.

The results for the parental/caregiver HRQoL demonstrated that the vast majority of participants (drawn from the general population) considered AADC deficiency to have a significant negative impact on individuals and families caring for these patients. This concurs with studies on other rare medical conditions which have highlighted the considerable caregiver burden both in terms of HRQoL, as well as household budgets [19, 20].

There are a number of potential study limitations that need to be addressed. The first one being the nature of the study itself drawing only from a panel of online participants. This, by definition, limits the sample to only those participants with access to the internet and could possibly limit the sample to younger respondents. However, this should be balanced against the fact that the sampling strategy was designed to ensure that at least a third of participants were aged 55 and above (in fact around 10% of the sample was aged 65 years and older). There was also an overrepresentation of females relative to the population (60% versus 40%), nevertheless this is almost certain to have been mitigated by the large sample sizes for both genders.

The second potential limitation being the incongruent responses defined, in this study, as higher utility values (on the TTO task) for the “worst” health state compared to the “best” health state, which affected about a fifth of responses. Inconsistencies of greater magnitude have been reported in the literature for both TTO and SG tasks [21, 22] and have been shown to be the result of a combination of task and respondent characteristics [23]. An analysis of the study results suggested that some sociodemographic differences had some influence on the logical consistency of responses. Furthermore, participants with logically inconsistent responses took less time to complete the study. However, although there was a non-statistically significant difference in the average time to completion, these participants were nevertheless taking on average around 11 min (633 s) to complete the study (compared to 811 or approximately 14 min for participants with consistent responses). A further post hoc logistic regression analysis was undertaken to evaluate potential predictors of incongruent responses which included gender (male/female), parental status (including parent of child < 16 years) (all as categorical variables), as well as completion time and education and age (continuous variable). Only parental status and gender were statistically significant predictors of incongruent responses in addition to completion time. The results indicated not being a parent (B =  − 0.41, standard error (SE) = 0.14, *p* = 0.003, odds ratio (OR) = 0.67 (95%CI 0.51 to 0.87)) and being female (B =  − 0.32, SE = 0.13, *p* = 0.013, OR = 0.73 (95%CI 0.56 to 0.94)) is less likely to be associated with incongruent responses (in other words being a parent and being a male is associated with incongruent responses). To further investigate incongruent responses a latent class analysis was undertaken using finite mixture models (FMM). In order to achieve this the total number of incongruent was calculated for each participant (up to a total of 10 combinations, i.e., bedridden > head control, head control > sitting, and so on) to create a new variable. FMM (linear regression model) were then derived both the whole sample (N = 1590) as well as the subset (N = 327) of participants with incongruent responses. Completion time, gender, parental status, education level and age were included as predictors. Two latent classes were fitted for both samples. For the whole sample, only completion time was a significant predictor for the incongruent response latent class only (*p* < 0.019). The estimated marginal means, i.e., mean number of incongruent responses was 2.14 (95%CI 2.06 to 2.21) compared to 0.82 (95%CI0.78 to 0.86) for the other latent class (Additional file 1: Table S1). The latent class marginal means were 0.45 and 0.55, respectively. Turning to the incongruent responses alone (Additional file 1: Table S2), within this sample one latent class was again characterised by shorter completion times (*p* = 0.014), as well as parental status (*p* = 0.031). The marginal mean for this class was 7.31 incongruent responses (CI95% 6.76 to 7.88) compared to 5.04 (95%CI 4.54 to 5.54) for the other latent class; the latent class marginal probabilities were 0.66 and 0.34 respectively. A final analysis was undertaken of those respondents categorized as congruent responders (N = 1293) (Additional file 1: Table S3). The vast majority of these respondents (79.3%) had completely congruent responses. The FMM analysis revealed no statistically significant differences for any of the predictor variables. In summary then, the class of incongruent responders appears to be characterised by shorter completion times; within this class of responders is a subset of participants who are parents. These results viewed collectively suggest that although participants were taking time to consider the vignettes, and may indeed have been considering their responses carefully, the study tasks, may have been a greater cognitive burden to them compared to those with logically consistent responses, and that this, along with gender and parental status, may have led to more incongruent responses. Finally, in terms of the incongruent responses, it should be noted that the average percentage of inconsistency was around 23% for all comparisons of adjacent health state utilities (for instance, “bedridden” compared to “head control”, “head control” compared to “sitting”, and so on). This is the percentage of inconsistencies when both the most obvious incongruent responses had been removed (“bedridden” > “walking with assistance” and the “non-walking states” > “walking with assistance (although the latter was already subsumed in the former). In other words, this reflects a high logical consistency rate in excess of 75%. It should be noted that “perfect” logical consistency for *all participants* is extremely unlikely, given variation in individual preferences and utility ratings between adjacent health states, in line with utility theory. These robust health state utilities for AADC deficiency may be viewed with a high degree of confidence in terms of use within economic models to evaluate interventions for this condition.

The range between the “bedridden” and “head control” health state utilities was relatively small for the SG task (0.01). This may indicate that participants had difficulty distinguishing between the poorest health states, although this was not observed on the TTO task (difference 0.043). Nevertheless, overall, for both tasks there was a very clear distinction between the “worst” and “best” health states (around 0.19 utilities for the SG task and 0.23 for the TTO) suggesting that participants were able to reflect the severity of the condition and distinguish between the health states.

A final possible limitation was the fact that the vignettes reflected global milestone and symptom improvement, i.e. improvement in one symptom as also associated with a reduction in severity of the other symptoms. This meant that it was not possible to identify the contribution of each symptom or milestone to the health state utilities. Although this was based on the literature [2], further work is needed to determine the exact drivers of the health state utilities.

## Conclusion

In conclusion, health state utilities were derived for AADC deficiency from a large sample size with incongruent responses removed and a high degree of logical consistency in the utility ordering of the health states. To the authors knowledge, this is the first study to derive health state utilities for AADC deficiency, and is, therefore by definition, also the largest study of its kind. Health state utilities increased as severity of symptoms decreased, and as various motor milestones improved. In addition to AADC deficiency, the results may also provide beneficial insights for deriving health (state) utilities in other rare conditions such as spinal muscular atrophy (SMA) where similar difficulties may be encountered [19]. For AADC deficiency, and particularly the health state utilities, further research is required to determine the exact drivers in terms of the symptoms and milestones impacting the most on the health state utilities. These robust data will be utilised in an economic evaluation of a gene therapy for AADC deficiency.

## Supplementary Information

**Additional file 1**: Supplementary Tables.

## Data Availability

Not applicable.

## Footnotes

1 A number of definitions could have been employed to describe incongruent responses. This definition was selected for clarity.
