# Supplementary material for: Eliciting health state utilities for Aromatic L-amino Acid Decarboxylase (AADC) deficiency: a UK vignette study
Source: J Patient Rep Outcomes. 2021 Dec 11;5:130. doi: 10.1186/s41687-021-00403-0 (PMC8665907; doi:10.1186/s41687-021-00403-0)
Supplement: Supplementary file 1 — Additional file 1: Supplementary Tables. [file 41687_2021_403_MOESM1_ESM.docx]

**Supplementary Table 1 – Finite Mixture Model analysis (whole sample)**

Finite mixture model Number of obs = 1,590

Log likelihood = -1892.4853

------------------------------------------------------------------------------

| Coefficient Std. err. z P>|z| [95% conf. interval]

-------------+---------------------------------------------------------------

1.Class | (base outcome)

-------------+---------------------------------------------------------------

2.Class |

_cons | .2096925 .0726224 2.89 0.004 .0673551 .3520298

------------------------------------------------------------------------------

Class: 1

Response: FMM

Model: regress

-----------------------------------------------------------------------------------

| Coefficient Std. err. z P>|z| [95% conf. interval]

------------------+---------------------------------------------------------------

FMM |

Durationinseconds | -.0000883 .0000375 -2.35 0.019 -.0001618 -.0000148

Age_1 | -.0005257 .0013984 -0.38 0.707 -.0032665 .0022151

Gender2 | .0044424 .0457713 0.10 0.923 -.0852676 .0941525

Education2 | -.0523309 .0334955 -1.56 0.118 -.1179809 .013319

IsParent2 | .0747181 .0493776 1.51 0.130 -.0220603 .1714964

_cons | 2.21685 .1327621 16.70 0.000 1.956641 2.477058

------------------+---------------------------------------------------------------

var(e.FMM)| .2461376 .0309608 .1923572 .3149544

-----------------------------------------------------------------------------------

Class: 2

Response: FMM

Model: regress

-----------------------------------------------------------------------------------

| Coefficient Std. err. z P>|z| [95% conf. interval]

------------------+---------------------------------------------------------------

FMM |

Durationinseconds | -.0000489 .0000328 -1.49 0.137 -.0001133 .0000155

Age_1 | -.0007795 .0011559 -0.67 0.500 -.003045 .001486

Gender2 | .0065012 .0363207 0.18 0.858 -.0646861 .0776884

Education2 | -.0436768 .0264679 -1.65 0.099 -.0955529 .0081993

IsParent2 | -.0348652 .0409821 -0.85 0.395 -.1151886 .0454583

_cons | 1.006942 .1049981 9.59 0.000 .8011499 1.212735

------------------+---------------------------------------------------------------

var(e.FMM)| .2054468 .0175145 .1738337 .2428091

-----------------------------------------------------------------------------------

. estat lcmean

Latent class marginal means Number of obs = 1,590

------------------------------------------------------------------------------

| Delta-method

| Margin std. err. z P>|z| [95% conf. interval]

-------------+---------------------------------------------------------------

1 |

FMM | 2.135461 .0364535 58.58 0.000 2.064013 2.206908

-------------+---------------------------------------------------------------

2 |

FMM | .8213436 .0206385 39.80 0.000 .780893 .8617943

------------------------------------------------------------------------------

. estat lcprob

Latent class marginal probabilities Number of obs = 1,590

--------------------------------------------------------------

| Delta-method

| Margin std. err. [95% conf. interval]

-------------+-----------------------------------------------

Class |

1 | .4477681 .0179575 .4128903 .4831676

2 | .5522319 .0179575 .5168324 .5871097

--------------------------------------------------------------

**Supplementary Table 2 – Finite Mixture Model analysis (incongruent responses only)**

Finite mixture model Number of obs = 327

Log likelihood = -566.95707

------------------------------------------------------------------------------

| Coefficient Std. err. z P>|z| [95% conf. interval]

-------------+---------------------------------------------------------------

1.Class | (base outcome)

-------------+---------------------------------------------------------------

2.Class |

_cons | -.6663144 .5340122 -1.25 0.212 -1.712959 .3803302

------------------------------------------------------------------------------

Class: 1

Response: FMM2

Model: regress

-----------------------------------------------------------------------------------

| Coefficient Std. err. z P>|z| [95% conf. interval]

------------------+---------------------------------------------------------------

FMM2 |

Durationinseconds | -.0002523 .0001027 -2.46 0.014 -.0004537 -.0000509

Age_1 | -.0012092 .0054147 -0.22 0.823 -.0118219 .0094034

Gender2 | -.146554 .204168 -0.72 0.473 -.546716 .253608

Education2 | .1277206 .1553008 0.82 0.411 -.1766634 .4321046

IsParent2 | -.4105391 .1904331 -2.16 0.031 -.7837811 -.0372971

_cons | 8.070135 .596965 13.52 0.000 6.900105 9.240165

------------------+---------------------------------------------------------------

var(e.FMM2)| 1.088075 .3338569 .5963246 1.985342

-----------------------------------------------------------------------------------

Class: 2

Response: FMM2

Model: regress

-----------------------------------------------------------------------------------

| Coefficient Std. err. z P>|z| [95% conf. interval]

------------------+---------------------------------------------------------------

FMM2 |

Durationinseconds | -.0002676 .0002281 -1.17 0.241 -.0007147 .0001796

Age_1 | -.0001015 .0056519 -0.02 0.986 -.011179 .0109761

Gender2 | -.1769914 .1822064 -0.97 0.331 -.5341094 .1801267

Education2 | -.1110659 .1090875 -1.02 0.309 -.3248736 .1027418

IsParent2 | -.0044893 .1901656 -0.02 0.981 -.377207 .3682284

_cons | 5.679205 .581289 9.77 0.000 4.539899 6.81851

------------------+---------------------------------------------------------------

var(e.FMM2)| .4677758 .1808916 .2192165 .998165

-----------------------------------------------------------------------------------

. estat lcmean

Latent class marginal means Number of obs = 327

------------------------------------------------------------------------------

| Delta-method

| Margin std. err. z P>|z| [95% conf. interval]

-------------+---------------------------------------------------------------

1 |

FMM2 | 7.318938 .2860971 25.58 0.000 6.758198 7.879678

-------------+---------------------------------------------------------------

2 |

FMM2 | 5.039312 .2548002 19.78 0.000 4.539913 5.538711

------------------------------------------------------------------------------

. estat lcprob

Latent class marginal probabilities Number of obs = 327

--------------------------------------------------------------

| Delta-method

| Margin std. err. [95% conf. interval]

-------------+-----------------------------------------------

Class |

1 | .6606774 .1197163 .4060473 .8472197

2 | .3393226 .1197163 .1527803 .5939527

--------------------------------------------------------------

**Supplementary Table 3 – Finite Mixture Model analysis (Congruent responses only)**

Finite mixture model Number of obs = 1,263

Log likelihood = -1253.9884

------------------------------------------------------------------------------

| Coefficient Std. err. z P>|z| [95% conf. interval]

-------------+----------------------------------------------------------------

1.Class | (base outcome)

-------------+----------------------------------------------------------------

2.Class |

_cons | -.8356795 .0631823 -13.23 0.000 -.9595146 -.7118444

------------------------------------------------------------------------------

Class: 1

Response: FMM

Model: regress

-----------------------------------------------------------------------------------

| Coefficient Std. err. z P>|z| [95% conf. interval]

------------------+----------------------------------------------------------------

FMM |

Gender2 | -.0080432 .0307986 -0.26 0.794 -.0684073 .0523209

IsParent2 | -.042814 .0350015 -1.22 0.221 -.1114157 .0257877

Education2 | -.032523 .0224139 -1.45 0.147 -.0764534 .0114075

Durationinseconds | -.0000256 .0000233 -1.10 0.273 -.0000713 .0000201

_cons | .9473174 .0782311 12.11 0.000 .7939873 1.100647

------------------+----------------------------------------------------------------

var(e.FMM)| .1804472 .010425 .1611289 .2020817

-----------------------------------------------------------------------------------

Class: 2

Response: FMM

Model: regress

-----------------------------------------------------------------------------------

| Coefficient Std. err. z P>|z| [95% conf. interval]

------------------+----------------------------------------------------------------

FMM |

Gender2 | -.0397039 .0276849 -1.43 0.152 -.0939653 .0145575

IsParent2 | .0301889 .0298391 1.01 0.312 -.0282946 .0886724

Education2 | -.024621 .019902 -1.24 0.216 -.0636282 .0143862

Durationinseconds | -.0000425 .0000257 -1.65 0.099 -.0000929 7.98e-06

_cons | 2.166789 .069269 31.28 0.000 2.031024 2.302554

------------------+----------------------------------------------------------------

var(e.FMM)| .0692043 .00519 .0597443 .0801621

-----------------------------------------------------------------------------------
